# New Drugs, Appliances, and Things Medical

**Published:** 1890-02-22

**Authors:** 


### NEW DRUGS, APPLIANCES, AND THINGS MEDICAL.

[All preparations, appliances, novelties, etc., of which a notice is desired, should be sent to The Editor, The Lodge, Porchester Square, W.]

### VAN HOUTEN'S COCOA.

We have received a parcel of the above for examination and find it to be a most excellent article. Microscopically it shows absence of starch or other added ingredients: and the average of several "fields" demonstrates the uniform character of the product. On estimating the amount of cocoa-butter present we find that all excess has been removed, thus obviating a well-known cause of dyspepsia in those who prefer cocoa to tea or coffee. Examined chemically for free starch and sugar none were found; nor after boiling with dilute acid was there a great amount of copper-reducing product found. It is claimed for this cocoa that it is very soluble. We tried the experiment with ordinary London water. Boiling a weighed quantity in a measured amount of water, it was noted that after 48 hours' rest the deposit of insoluble residue was very small. Decanting the solution from the residue, the latter was again boiled in excess of water, and found to be nearly all dissolved. This cocoa is therefore most soluble, easily digestible and delicate in flavour. We can thoroughly recommend Van Houten's Cocoa as a wholesome drink and pleasant food.

### TUCKER'S EUCALYPTUS PREPARATIONS.

We have received various specimens of the above firm's manufacture from their stores in Paddington Street. Messrs. Tucker have applied eucalyptus products not only to medical, surgical, and sanitary purposes, but also to those of the toilet. Among the latter we note very pleasant forms of hair wash, toilet vinegar, tooth powder, and liquid dentifrice and a soap. The firm's more medical preparations are well made; they include a long list of disinfectant and antiseptic materials having the eucalyptus oil as the active ingredient. Amongst them are a powder, fluid (we tried this lately in a case of diphtheria, and it answered well), spray, and various ointments and emulsions, made after the formulæ of Sir Joseph Lister. Mr. Curgenven has recently, in the *British Medical Journal*, drawn attention to the use of eucalyptus as a disinfectant and protective in scarlet fever, and also as a deodorant in cases of foul discharges, as in cancer. He speaks highly of Messrs. Tucker's "disinfectant" fluid, which we believe is chiefly composed of thymol dissolved in oil of eucalyptus. We can corroborate his evidence as to the usefulness of this form of antiseptic. The advantages of eucalyptus as an antiseptic are: first, it is non-poisonous; secondly, it is a very powerful germicide; thirdly, it is easy of application; fourthly, it is a deodorant as well as an antiseptic. Its chief disadvantage does not concern its domestic use. It is its volatility; it was found that the gauzes for surgical use did not retain their relative strength after a time; this was due to loss of the eucalyptus oil by evaporation. Some years ago we had considerable experience of the use of eucalyptus oil in a large outbreak of diphtheria, and were most favourably impressed with its power of relief. Since then we have always used it, either alone or in conjunction with other remedies. A bronchitis kettle well charged with eucalyptus gives most decided relief in the throat symptoms of scarlet and other fevers.

